# Evaluation of the 2020 Pediatric Emergency Physician Workforce in the US

**DOI:** 10.1001/jamanetworkopen.2021.10084

**Published:** 2021-05-18

**Authors:** Christopher L. Bennett, Janice A. Espinola, Ashley F. Sullivan, Krislyn M. Boggs, Carson E. Clay, Moon O. Lee, Margaret E. Samuels-Kalow, Carlos A. Camargo

**Affiliations:** 1 Department of Emergency Medicine, Stanford University School of Medicine, Stanford, California; 2 Emergency Medicine Network, Department of Emergency Medicine, Massachusetts General Hospital, Harvard Medical School, Boston, Massachusetts

## Abstract

**Importance:**

Given the mortality disparities among children and adolescents in rural vs urban areas, the unique health care needs of pediatric patients, and the annual emergency department volume for this patient population, understanding the availability of pediatric emergency physicians (EPs) is important. Information regarding the available pediatric EP workforce is limited, however.

**Objective:**

To describe the demographic characteristics, training, board certification, and geographic distribution of the 2020 clinically active pediatric EP workforce in the US.

**Design, Setting, and Participants:**

This national cross-sectional study of the 2020 pediatric EP workforce used the American Medical Association Physician Masterfile database, which was linked to American Board of Medical Specialties board certification information. Self-reported training data in the database were analyzed to identify clinically active physicians who self-reported pediatric emergency medicine (EM) as their primary or secondary specialty. The Physician Masterfile data were obtained on March 11, 2020.

**Main Outcomes and Measures:**

The Physician Masterfile was used to identify all clinically active pediatric EPs in the US. The definition of EM training was completion of an EM program (inclusive of both an EM residency and/or a pediatric EM fellowship) or a combined EM program (internal medicine and EM, family medicine and EM, or pediatrics and EM). Physician location was linked and classified by county-level Urban Influence Codes. Pediatric EP density was calculated and mapped using US Census Bureau population estimates.

**Results:**

A total of 2403 clinically active pediatric EPs were working in 2020 (5% of all clinically active emergency physicians), of whom 1357 were women (56%) and the median (interquartile range) age was 46 (40-55) years. The overall pediatric EP population included 1718 physicians (71%) with EM training and 641 (27%) with pediatric training. Overall, 1639 (68%) were board certified in pediatric EM, of whom 1219 (74%) reported EM training and 400 (24%) reported pediatrics training. Nearly all pediatric EPs worked in urban areas (2369 of 2402 [99%]), and pediatric EPs in urban compared with rural areas were younger (median [interquartile range] age, 46 [40-55] years vs 59 [48-65] years). Pediatric EPs who completed their training 20 years ago or more compared with those who completed training more recently were less likely to work in urban settings (633 [97%] vs 0-4 years: 440 [99%], 5-9 years: 547 [99%], or 10-19 years: 723 [99%]; *P* = .006). Three states had 0 pediatric EPs (Montana, South Dakota, and Wyoming), and 3 states had pediatric EPs in only 1 county (Alaska, New Mexico, and North Dakota). Less than 1% of counties had 4 or more pediatric EPs per 100 000 population.

**Conclusions and Relevance:**

This study found that almost all pediatric EPs worked in urban areas, leaving rural areas of the US with limited availability of pediatric emergency care. This finding may have profound implications for children and adolescents needing emergency care.

## Key Points

**Question:**

What are the characteristics of the 2020 pediatric emergency physician workforce, and where do these physicians practice?

**Findings:**

In this cross-sectional study of 2403 self-identified pediatric emergency physicians in the US workforce, 99% reported working in urban areas. Three states had no pediatric emergency physicians (Montana, South Dakota, and Wyoming), 3 states had pediatric emergency physicians in only 1 county (Alaska, New Mexico, and North Dakota), and 17 counties (<1% of all counties) had 4 or more pediatric emergency physicians per 100 000 population.

**Meaning:**

Results of this study suggest that vast areas of the US lack availability of pediatric emergency physicians.

## Teaser

This cross-sectional study reviews the self-reported demographic characteristics, geographic distribution, as well as board certification and training of US-based clinically active physicians who provide emergency care to children and adolescents.

## Introduction

Children have unique health care needs that are critical to address in times of health-related emergencies and in the setting of chronic, complex medical conditions.^1^ Given that the leading causes of death for children and adolescents in the US are motor vehicle collisions, firearm-related injuries, and malignant neoplasms, emergency departments (EDs) play a vital role in the emergency care of these younger patients.^2^ Children and adolescents account for roughly one-quarter of all ED visits in the US.^3^ However, most of these patients are seen not in specialty (stand-alone) children’s hospitals but in general EDs with variable pediatric capabilities^4^ and limited pediatric readiness.^5,6,7,8,9^ Most emergent care for pediatric patients in the US is also provided by emergency physicians (EPs) without specialized training in pediatric emergency medicine (EM).^10,11^ Although the emergent care of pediatric patients is well within the scope of practice for physicians with training and board certification in general EM, children and adolescents have different clinical needs (compared with adults) and some EPs have decreased comfort caring for this population.^7,12^

In the US, many rural areas have limited or no EP availability,^13,14^ and EPs who specialize in the emergency care of pediatric patients appear to be even more scarce.^13,14^ Furthermore, disparities in mortality patterns among children and adolescents exist; children and adolescents in rural areas have higher mortality than their suburban and urban peers.^2^ Given these disparities, the unique health care needs of pediatric patients, and the annual ED volume for this population, understanding the availability of pediatric EPs is important. Lack of availability of pediatric EPs may be an important barrier to high-quality, specialized pediatric emergency care.

A recent study performed a comprehensive analysis of the clinically active EP workforce in the US, and it described the current demographic characteristics, self-reported training, board certification status, and geographic distribution of all clinically active EPs.^13^ The study found that most clinically active EPs were trained or board certified in EM. Although the work demonstrated an increase in the total population of EPs, it concurrently found decreased presence in rural areas, including a band of rural states, from North Dakota to Texas, with markedly fewer EPs.^13^ To our knowledge, no similar study exists that focuses on the pediatric EP workforce. Such a study could provide a benchmark for the current pediatric EP workforce and could further serve as a means to identify areas within the US that are underserved by, or completely lacking, pediatric EPs. These areas could be targeted with new approaches (eg, telepediatrics)^15^ to improve the availability of specialized pediatric EP care where it is needed most.

Given the need for such information, we completed a comprehensive study of the 2020 clinically active pediatric EP workforce in the US. Our primary objective was to describe the demographic characteristics, training, board certification, and geographic distribution of these physicians. Informed by the findings of a previous study of the overall EP population,^13^ we hypothesized that rural areas would have a markedly decreased availability of pediatric EPs.

## Methods

### Data Source and Design

We conducted a national cross-sectional study of the pediatric EP workforce using the 2020 American Medical Association (AMA) Physician Masterfile database.^16^ As previously described,^13,14^ this database is a comprehensive resource that includes data on more than 1.4 million physicians, residents, and medical students. Established by the AMA in 1906 as a record-keeping device, the Physician Masterfile is a data set that has been used to study the physician workforce in recent years, including previous and current research by our group. The Physician Masterfile data version used in this analysis was obtained on March 11, 2020. The study protocol was deemed exempt from review by the Mass General Brigham (formerly Partner’s Healthcare) Institutional Review Board, which waived informed consent because the work was a secondary review of already obtained, deidentified data. We followed the Strengthening the Reporting of Observational Studies in Epidemiology (STROBE) reporting guideline.^17^

### Outcome Measures and Variables

To identify all clinically active pediatric EPs in the US, we used self-designated practice specialties or areas of practice (SDPS) pediatric EM codes listed in the Physician Masterfile (EMP [pediatrics-emergency medicine], PE [pediatric emergency medicine-EM], and PEM [pediatric emergency medicine]). The database reflects physicians at the time data were obtained and provides information on physician location and self-reported sex. As of March 11, 2020,^13^ the database contained 3525 physicians with SDPS codes corresponding to a primary or secondary specialty of pediatric EM. Given that the focus of this study was on clinically active physicians, we followed the approach of previously published studies^13,14^ and excluded resident physicians (n = 551); physicians who identified as primarily in administrative (n = 39), teaching (n = 79), research (n = 15), or nonclinical (n = 4) fields; those who were retired or semiretired (n = 66); those who were temporarily not in practice (n = 6); and those who were not active for other reasons (n = 4). We also excluded physicians who did not provide classification information about their clinical status (n = 358). Included physicians were self-identified, clinically active pediatric EPs who met the inclusion criteria.^13^ As previously described,^13^ variables were self-reported and defined by the physicians at the time of either license application or renewal. In addition to physician specialty (both primary and secondary), location and sex were also self-reported.

In line with previous work,^13,14^ for the purpose of this study we assumed that physicians lived and worked in the same county. The approach to US Census Bureau divisions was also conserved; location was classified into the same 9 census divisions as those used in previous studies (New England, Mid-Atlantic, East North Central, West North Central, South Atlantic, East South Central, West South Central, Mountain, and Pacific).^13,14^ We also used the same metropolitan statistical area classification system for population sizes and Urban Influence Codes to categorize urban (categories 1 and 2) and rural (categories 3-12) areas.^18^ We used completion of an Accreditation Council for Graduate Medical Education (ACGME) program to classify training.

We used this intentionally inclusive definition of EM training: completion of an ACGME-accredited EM program (inclusive of both an EM residency and/or a pediatric EM fellowship) or a combined EM program (internal medicine and EM, family medicine and EM, or pediatrics and EM). In line with previous work,^13,14^ we also classified training for physicians without EM training as follows: family medicine, internal medicine, pediatrics, surgery, and other. In the context of this work, pediatrics training referred to no additional training beyond a pediatrics residency; EM training was inclusive of pediatric EPs with any component of EM training, including a pediatric EM fellowship. As in the previous study,^13^ we listed transitional year separately from preliminary internship; those who completed only an internship were classified as internship only. Years since training was based on the graduation year from the most recently completed training program. In addition, we linked to the American Board of Medical Specialties for board certification information (both general EM and pediatric EM). For analysis, pediatric EPs with board certification in both pediatric EM and general EM were included in the pediatric EM board certification group. Overall, missing data were rare (predominately metropolitan statistical area information) and were not imputed.

### Statistical Analysis

We evaluated the pediatric EP population using Stata, version 14.2 (StataCorp LLC). Data were summarized with descriptive statistics, including medians with interquartile range (IQR) and proportions. As in the previous study,^13^ we did not report CIs given that the AMA Physician Masterfile is an inclusive resource that represents the total population of EPs. However, we compared stratified groups of pediatric EPs using χ^2^, Fisher exact test, and Kruskal-Wallis test, as appropriate. All *P* values were 2-tailed, with *P* < .05 considered to be statistically significant. We used US Census Bureau–based county-level resident population estimates for 2019 to calculate physician density (defined as number of EPs per 100 000 population). We created EP population density maps by county using ArcMap 10.6.1 (Esri).

## Results

We identified 2403 clinically active pediatric EPs in 2020 who reported pediatric EM as their primary or secondary specialty, of whom 1952 (81%) reported pediatric EM as their primary specialty; a total of 1122 pediatric EPs were excluded. The overall pediatric EP population (median [IQR] age, 46 [40-55] years; 1357 women [56%] and 1046 men [44%]) represents 5% of the overall clinically active EP population.^13^ The characteristics of these physicians, both overall and by board certification, are presented in Table 1.

**Table 1.** Characteristics of Pediatric Emergency Physicians by Board Certification

Characteristic	Overall, No. (%)	EM board certification, No. (%)			No board certification, No. (%)	*P* value
		Pediatric^a^	General	Neither pediatric nor general		
No. of physicians	2403 (100)	1639 (68)	103 (4)	568 (24)	93 (4)	
**Demographic**						
Age, median (IQR), y	46 (40-55)	47 (42-56)	42 (38-50)	40 (36-54)	43 (36-55)	<.001
Age categories, y						
25-44	1086 (45)	636 (39)	65 (63)	332 (58)	53 (57)	<.001
45-64	1162 (48)	905 (55)	33 (32)	193 (34)	31 (33)	
≥65	155 (6)	98 (6)	5 (5)	43 (8)	9 (10)	
Female sex	1357 (56)	923 (56)	49 (48)	323 (57)	62 (67)	.06
IMG	443 (18)	262 (16)	8 (8)	148 (26)	25 (27)	<.001
**Geographic**						
US census division						
New England	182 (8)	126 (8)	10 (10)	40 (7)	6 (7)	<.001
Mid-Atlantic	406 (17)	303 (19)	12 (12)	80 (14)	11 (13)	
East North Central	318 (13)	207 (13)	20 (20)	80 (14)	11 (13)	
West North Central	145 (6)	100 (6)	3 (3)	36 (6)	6 (7)	
South Atlantic	523 (22)	349 (21)	16 (16)	137 (24)	21 (24)	
East South Central	129 (5)	89 (5)	2 (2)	36 (6)	2 (2)	
West South Central	242 (10)	161 (10)	9 (9)	68 (12)	4 (5)	
Mountain	186 (8)	108 (7)	19 (19)	49 (9)	10 (11)	
Pacific	262 (11)	194 (12)	11 (11)	41 (7)	16 (18)	
MSA population size						
<100 000	3 (0.1)	2 (0.1)	0	1 (0.2)	0	<.001
100 000-249 999	45 (2)	27 (2)	7 (7)	9 (2)	2 (2)	
250 000-999 999	284 (12)	179 (11)	19 (18)	74 (13)	12 (13)	
≥1 000 000	2019 (84)	1408 (86)	69 (67)	463 (82)	79 (85)	
Unknown	52 (2)	23 (1)	8 (8)	21 (4)	0	
Physician location						
Urban	2369 (99)	1622 (99)	97 (94)	557 (98)	93 (100)	.01
Large rural	26 (1)	13 (0.8)	5 (5)	8 (1)	0	
Small rural	7 (0.3)	3 (0.2)	1 (1)	3 (0.5)	0	
**Training**						
EM^b^	1718 (71)	1219 (74)	97 (94)	341 (60)	61 (66)	<.001
Family medicine	0	0	0	0	0	
Internal medicine	15 (0.6)	6 (0.4)	0	9 (2)	0	
Pediatrics^b^	641 (27)	400 (24)	3 (3)	215 (38)	23 (25)	
Surgery	2 (0)	0	0	0	2 (2)	
Internship only	1 (0)	0)	1 (1)	0	0	
Other	0	0	0	0)	0	
None	26 (1)	14 (0.9)	2 (2)	3 (0.5)	7 (8)	
Years since completion of training						
<5	443 (19)	89 (5)	28 (28)	284 (50)	42 (49)	<.001
5-9	551 (23)	460 (28)	28 (28)	54 (10)	9 (10)	
10-19	730 (31)	608 (37)	32 (32)	75 (13)	15 (17)	
≥20	653 (27)	468 (29)	13 (13)	152 (27)	20 (23)	
Primary specialty, pediatric EM	1952 (81)	1359 (83)	82 (80)	436 (77)	75 (81)	.01

Abbreviations: EM, emergency medicine; IMG, international medical graduate; IQR, interquartile range; MSA, metropolitan statistical area.

^a^ For analysis, pediatric emergency physicians who were board certified in both pediatric EM and general EM (n = 186) were included in the pediatric EM board certification group.

^b^ Training in EM was defined as completion of an EM program (inclusive of EM residency and pediatric EM fellowship) or a combined EM program (internal medicine and EM, family medicine and EM, and pediatrics and EM).

Of the overall pediatric EP population, 1718 physicians (71%) reported previous EM training and 641 (27%) had previous pediatrics training. A total of 103 physicians (4%) were board certified in general EM, 1639 (68%) were board certified in pediatric EM (of this group, 1219 [74%] reported EM training and 400 [24%] reported pediatrics training), 568 (24%) were board certified but not in either general or pediatric EM, and 93 (4%) had no board certification. A subset of pediatric EPs were board certified in both pediatric EM and general EM (n = 186). With a median (IQR) age of 47 (42-56) years, pediatric EPs with pediatric EM board certification were older than pediatric EPs with general EM board certification (42 [38-50] years), those without either general or pediatric EM board certification (40 [36-54] years), and those without any board certification (43 [36-55] years).

International medical graduates were less likely to be board certified in general EM (8 [8%]) or pediatric EM (262 [16%]) and more likely to have neither general nor pediatric EM board certification (148 [26%]) or no board certification at all (25 [27%]). The most common training pathways were EM for pediatric EPs without either general or pediatric EM board certification (341 [60%]) or without any board certification (61 [66%]) and pediatrics for those without general or pediatric EM board certification (215 [38%]) or without board certification (23 [25%]). Characteristics of pediatric EPs with pediatric EM board certification, stratified by either EM training or isolated pediatrics training, are presented in the eTable in the Supplement.

Table 2 presents characteristics of pediatric EPs by rural vs urban areas. Nearly all pediatric EPs (2369 of 2402 [99%]) worked in urban areas, with only 33 (1%) serving rural areas. With a median (IQR) age of 59 (48-65) years, pediatric EPs in rural areas were older than their urban counterparts who had a median (IQR) age of 46 (40-55) years. Furthermore, pediatric EPs in rural areas compared with those in urban areas were more likely to have completed medical training 20 years ago or more (20 [61%] vs 633 [27%]; *P* < .001), were less likely to have pediatric EM board certification (16 [48%] vs 1622 [68%]; *P* = .01), and were more likely to be board certified in another specialty (17 [52%] vs 654 [28%]; *P* = .01).

**Table 2.** Comparison of Pediatric Emergency Physicians in Urban vs Rural Areas^a^

Characteristic	No. (%)			*P* value
	Overall	Urban area	Rural area	
No. of physicians	2402 (100)	2369 (99)	33 (1)	
**Demographic**				
Age, median (IQR), y	46 (40-55)	46 (40-55)	59 (48-65)	<.001
Age categories, y				
25-44	1085 (45)	1080 (46)	5 (15)	<.001
45-64	1162 (48)	1143 (48)	19 (58)	
≥65	155 (6)	146 (6)	9 (27)	
Female sex	1356 (56)	1337 (56)	19 (58)	.90
IMG	442 (18)	440 (19)	2 (6)	.07
**Geographic**				
US census division				
New England	182 (8)	177 (8)	5 (15)	.02
Mid-Atlantic	406 (17)	405 (17)	1 (3)	
East North Central	318 (13)	315 (13)	3 (9)	
West North Central	145 (6)	145 (6)	0	
South Atlantic	523 (22)	519 (22)	4 (12)	
East South Central	129 (5)	127 (5)	2 (6)	
West South Central	242 (10)	235 (10)	7 (21)	
Mountain	186 (8)	181 (8)	5 (15)	
Pacific	262 (11)	256 (11)	6 (18)	
MSA population size				
<100 000	3	3	0	<.001
100 000-249 999	45 (2)	45 (2)	0	
250 000-999 999	284 (12)	282 (12)	2 (6)	
≥1 000 000	2019 (84)	2018 (85)	1 (3)	
Unknown	51 (2)	21 (1)	30 (91	
**Training**				
EM	1717 (71)	1699 (72)	18 (55)	.17
Family medicine	0	0	0	
Internal medicine	15 (1)	15 (1)	0	
Pediatrics	641 (27)	626 (26)	15 (45)	
Surgery	2 (0)	2 (0)	0	
Internship only	1 (0)	1 (0)	0	
Other	0	0	0	
None	26 (1)	26 (1)	0	
Years since completion of training				
<5	443 (19)	440 (19)	3 (9)	<.001
5-9	551 (23)	547 (23)	4 (12)	
10-19	729 (31)	723 (31)	6 (18)	
≥20	653 (27)	633 (27)	20 (61)	
Primary specialty, pediatric EM	1951 (81)	1934 (82)	17 (52)	<.001
**Board certification**				
Pediatric EM	1638 (68)	1622 (68)	16 (48)	.01
Other specialty	671 (28)	654 (28)	17 (52)	
None	93 (4)	93 (4)	0	

Abbreviations: EM, emergency medicine; IMG, international medical graduate; IQR, interquartile range; MSA, metropolitan statistical area.

^a^ One of 2403 pediatric emergency physicians (.04%) excluded because of missing county data needed for urbanicity classification.

Table 3 presents characteristics of pediatric EPs by years since completion of training. Pediatric EPs who completed their training 20 years ago or more compared with those who completed training more recently were more likely to be international medical graduates (164 [25%] vs 0-4 years: 86 [19%], 5-9 years: 85 [15%], or 10-19 years: 99 [14%]; *P* < .001), less likely to be women (302 [46%] vs 0-4 years: 279 [63%], 5-9 years: 343 [62%], or 10-19 years: 420 [58%]; *P* < .001), less likely to have EM training (92 [14%] vs 0-4 years: 437 [99%], 5-9 years: 529 [96%], or 10-19 years: 660 [90%]; *P* < .001) or pediatric EM board certification (468 [72%] vs 0-4 years: 89 [20%], 5-9 years: 460 [83%], or 10-19 years: 608 [83%]; *P* < .001), and less likely to work in urban settings (633 [97%] vs 0-4 years: 440 [99%], 5-9 years: 547 [99%], or 10-19 years: 723 [99%]; *P* = .006).

**Table 3.** Characteristics of Pediatric Emergency Physicians by Years Since Completion of Training^a^

Characteristic	Years since training graduation, No. (%)				*P* value
	0-4 y	5-9 y	10-19 y	≥20 y	
No. of physicians	443	551	730	653	
**Demographic**					
Age, median (IQR), y	36 (35-38)	41 (39-43)	47 (45-50)	60 (57-64)	<.001
Age categories, y					<.001
25-44	429 (97)	483 (88)	172 (24)	0	
45-64	14 (3)	68 (12)	553 (76)	510 (78)	
≥65	0	0	5 (1)	143 (22)	
Sex					
Female	279 (63)	343 (62)	420 (58)	302 (46)	<.001
Male	164 (37)	208 (38)	310 (42)	351 (54)	
IMG	86 (19)	85 (15)	99 (14)	164 (25)	<.001
**Geographic**					
US census division					
New England	39 (9)	45 (8)	62 (9)	33 (5)	.006
Mid-Atlantic	56 (13)	83 (15)	125 (17)	139 (21)	
East North Central	72 (16)	73 (13)	92 (13)	80 (12)	
West North Central	34 (8)	25 (5)	40 (6)	44 (7)	
South Atlantic	93 (21)	115 (21)	151 (21)	155 (24)	
East South Central	26 (6)	29 (5)	39 (5)	33 (5)	
West South Central	50 (11)	60 (11)	74 (10)	57 (9)	
Mountain	37 (8)	53 (10)	52 (7)	43 (7)	
Pacific	35 (8)	68 (12)	92 (13)	65 (10)	
MSA population size					
<100 000	1 (0.2)	1 (0.2)	1 (0.1)	0	.02
100 000-249 999	13 (3)	7 (1)	14 (2)	10 (2)	
250 000-999 999	48 (11)	70 (13)	79 (11)	82 (13)	
≥1 000 000	373 (84)	468 (85)	624 (85)	534 (82)	
Unknown	8 (2)	5 (0.9)	12 (2)	27 (4)	
Urban influence					
Urban	440 (99)	547 (99)	723 (99)	633 (97	.006
Large rural	3 (0.7)	3 (0.5)	4 (0.6)	16 (2)	
Small rural	0	1 (0.2)	2 (0.3)	4 (0.6)	
**Training**					
EM	437 (99)	529 (96)	660 (90)	92 (14)	<.001
Family medicine	0	0	0	0	
Internal medicine	1 (0.2)	0	3 (0.4)	11 (2)	
Pediatrics	5 (1)	22 (4)	67 (9)	547 (84)	
Surgery	0	0	0	2 (0.3)	
Internship only	0	0	0	1 (0.2)	
Other	0	0	0	0	
Primary specialty, pediatric EM	443 (100)	546 (99)	649 (89)	297 (45)	<.001
**Board certification**					
Pediatric EM	89 (20)	460 (83)	608 (83)	468 (72)	<.001
Other specialty	312 (70)	82 (15)	107 (15)	165 (25)	
None	42 (9)	9 (2)	15 (2)	20 (3)	

Abbreviations: EM, emergency medicine; IMG, international medical graduate; IQR, interquartile range; MSA, metropolitan statistical area.

^a^ A total of 26 pediatric emergency physicians (1%) excluded because of missing previous training information.

Pediatric EP physician density per 100 000 population by county is presented in the Figure. In 2020, 3 states had 0 pediatric EPs (Montana, South Dakota, and Wyoming), and 3 states had pediatric EPs in only 1 county (Alaska, New Mexico, and North Dakota). Most US counties (2784 [87%]) had 0 pediatric EPs, and 17 counties (<1%) had 4 or more pediatric EPs per 100 000 population.

**Figure.**
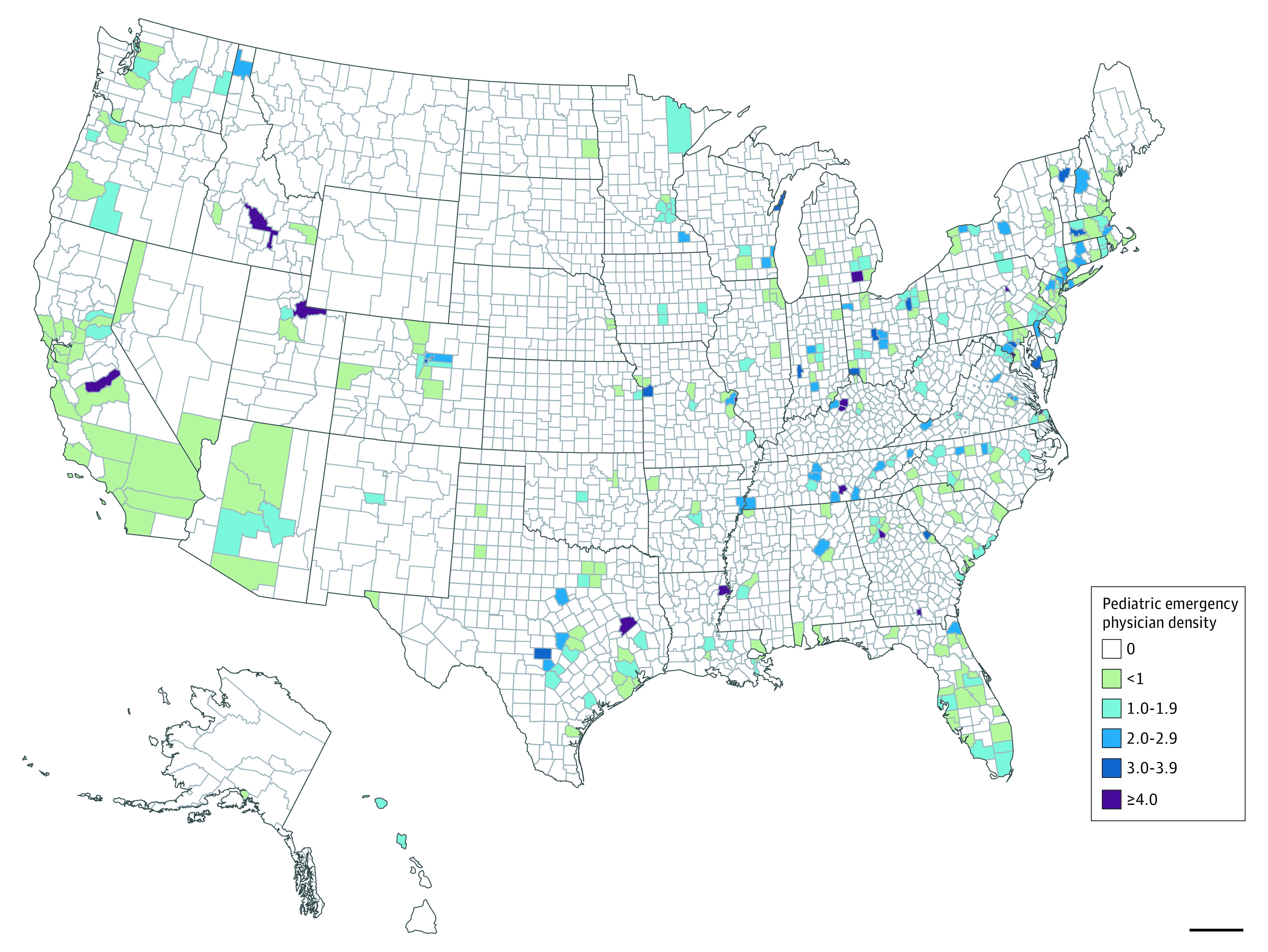
Pediatric Emergency Physician (EP) Density per 100 000 Population by County Three states had 0 pediatric EPs (Montana, South Dakota, and Wyoming), and 3 states had pediatric EPs in only 1 county (Alaska, New Mexico, and North Dakota). Most US counties (n = 2784) had 0 pediatric EPs per 100 000 population, 176 counties had fewer than 1, 107 counties had 1 to 1.9, 44 counties had 2 to 2.9, 14 counties had 3 to 3.9, and 17 counties had 4 or more.

## Discussion

Using the most complete available database on US physicians, we identified 2403 clinically active pediatric EPs in 2020. This total shows an increase of 1271 pediatric EPs since a 2008 national study on the overall EP workforce.^14^ To our knowledge, the current work is the most comprehensive analysis of a relatively small but essential part of the overall US emergency care workforce. More than half (68%) of pediatric EPs reported having pediatric EM board certification. Furthermore, 24% of physicians lacked any form of EM board certification and 4% had no board certification.

Ninety-nine percent of pediatric EPs worked in urban areas and, at their current numbers, cannot be expected to provide most pediatric emergency care. Meanwhile, pediatric EPs in rural areas compared with those in urban areas were significantly older and approaching the US retirement age of 66 years. These same rural pediatric EPs were also less likely to have pediatric EM board certification. Our mapping further revealed that pediatric EPs were not equally distributed across the US; some states had only 1 pediatric EP in the entire state, whereas other states had no pediatric EPs at all. Collectively, these findings suggest that, in the coming years, a group of rural pediatric EPs are poised to retire and leave the clinically active workforce. This workforce gap would further exacerbate the availability of pediatric EP specialist care in regions that are already vastly underserved.^13,14^ Without mitigation, this gap could have implications for the outcomes of children and adolescents seeking emergent care in rural areas, a population with a mortality rate that is already higher than that of its suburban and urban peers.^2^

The finding of unequal distribution of pediatric EPs is consistent with the results of recent research on the overall EP workforce, which similarly reported that a group of rural EPs were nearing the US retirement age.^13^ Despite an increase in the absolute number of EPs, decreases in EP density per 100 000 population in rural areas were noted over the past decade.^13^ Taken together, it is possible that as the older pediatric EPs retire and leave the workforce so too will the general EPs. In addition to facing limited or no availability of specialized pediatric EPs, many children seeking emergent care in rural areas will have limited or no access to any EPs. We also anticipate that pediatric patients will continue to make up a sizable proportion of the total annual ED visits in the US; thus, there could be an ongoing, unmet need for pediatric EPs. Absence of pediatric EPs (paired with absence of overall EPs) will likely accelerate the use of physician assistants and nurse practitioners^19,20^ and the increased use of alternative forms of emergency care, including telepediatrics.^21,22^

### Limitations

This study has several limitations. First, the AMA Physician Masterfile is the most comprehensive, frequently updated resource on US physicians, and inclusion in this database is independent of AMA membership (both members and nonmembers of the AMA are included). Although the AMA has dedicated staff to provide quality assurance for the Physician Masterfile, we cannot be certain of the frequency with which each of the individual data elements is updated. Much of the data presented here were self-reported, including physician training and primary or secondary SDPS pediatric EM codes. Furthermore, given the self-reported nature of SDPS codes, which has implications for all studies that use the Physician Masterfile, we were unable to verify which physicians completed or did not complete additional fellowship training (pediatric EM fellowship) beyond residency; this information, to our knowledge, does not exist.

Second, the self-reported physician location is also a limitation. As noted in the previous studies based on the Physician Masterfile,^13,14^ this analysis assumed that pediatric EPs lived and worked in the same county. As in previous studies,^13,14^ we were unable to account for the possible confounder of pediatric EPs living near county lines and practicing in nearby counties or the implications for the findings. In particular, locum EPs are physicians who often do not live and work in the same county, but no data set or resource on this small EP subpopulation is available, to our knowledge.^13^ Furthermore, as was the case in the previous work,^13^ the distribution of EP density by county demonstrated in this study (as well as low values seen in bands of states and in regions) argues against any substantial impact of locum EPs. Furthermore, use of Urban Influence Codes does limit the potential misclassification of rurality by accounting for adjacent commuting and economic centers of influence.^13,14^

Third, the Physician Masterfile data were linked to only ACGME-accredited training programs and American Board of Medical Specialties board certification information. This study, similar to the previous studies that used the Physician Masterfile,^13,14^ likely underestimated osteopathic physicians. Currently, no osteopathic pathways exist for pediatric EM board certification (either by the American Osteopathic Board of Emergency Medicine or the American Osteopathic Board of Pediatrics). However, physicians with American Osteopathic Board of Emergency Medicine board certification and who completed an ACGME-approved pediatric EM fellowship are eligible to take the American Board of Emergency Medicine board certification examination in pediatric EM.^23^ Consequently, the current analysis, with its linked American Board of Medical Specialties information, likely captured the subset of osteopathic physicians with pediatric EM board certification, representing all (allopathic and osteopathic) pediatric EPs with pediatric EM board certification. Regardless of these limitations, we believe this work presents the most comprehensive snapshot of clinically active physicians who currently provide emergency care to pediatric patients in the US.

## Conclusions

We believe that this study sheds light on the demographic characteristics, geographic distribution, board certification, and training of a relatively small but essential part of the overall US emergency care workforce: clinically active pediatric EPs. Given that nearly all pediatric EPs reported working in urban areas and rural pediatric EPs were older, we anticipate a worsening shortage of pediatric EPs in already underserved regions of the country, namely rural areas. We encourage the use of new approaches, such as telepediatrics, to improve access to high-quality pediatric emergency care in rural regions. The consequences for this specialty and for young patients are potentially profound.

## Notes

Supplement. **eTable.** Characteristics of Pediatric Emergency Physicians with Pediatric Emergency Medicine Board Certification by Training
