# Supplementary material for: Evaluation of the 2020 Pediatric Emergency Physician Workforce in the US
Source: JAMA Netw Open. 2021 May 18;4(5):e2110084. doi: 10.1001/jamanetworkopen.2021.10084 (PMC8132138; doi:10.1001/jamanetworkopen.2021.10084)
Supplement: Supplement. — eTable. Characteristics of Pediatric Emergency Physicians with Pediatric Emergency Medicine Board Certification by Training [file jamanetwopen-e2110084-s001.pdf]

Supplementary Online Content

Bennett CL, Espinola JA, Sullivan AF, et al. Evaluation of the 2020 pediatric emergency physician workforce in the US. *JAMA Netw Open*. 2021;4(5):e2110084. doi:10.1001/jamanetworkopen.2021.10084

eTable. Characteristics of Pediatric Emergency Physicians with Pediatric Emergency Medicine Board Certification by Training

This supplementary material has been provided by the authors to give readers additional information about their work.

© 2021 Bennett CL et al. *JAMA Network Open.*

eTable. Characteristics of Pediatric Emergency Physicians with Pediatric Emergency Medicine Board Certification by Training[a]

Characteristics	Pediatric Emergency Physicians with Pediatric Emergency Medicine Board Certification n=1,619 EM trained (n=1,219)		Pediatrics Trained, (n=400)		P-value
	n	%	n	%	
**Demographics**					
Age, y (median [IQR])	1,219	44 (41-49)	400	59 (56-63)	<0.001
Age categories, y					<0.001
25-44	634	52	2	1	
45-64	570	47	322	81	
≥65	15	1	76	19	
Female	721	59	195	49	<0.001
IMG	167	14	90	23	<0.001
**Geographic**					
**US Census division**					0.41
New England	98	8	27	7	
Mid Atlantic	209	17	90	23	
East North Central	158	13	49	12	
West North Central	69	6	29	7	
South Atlantic	264	22	80	20	
East South Central	66	5	21	5	
West South Central	122	10	38	10	
Mountain	84	7	23	6	
Pacific	147	12	43	11	
**MSA population size**					0.21
≥1,000,000	1,055	87	334	84	
250,000-999,999	126	10	52	13	
100,000-249,999	22	2	5	1	
<100,000	2	0	0	0	
Unknown	14	1	9	2	
**Physician Location**					0.15
Urban	1,208	99	394	99	
Large Rural	7	0.6	6	2	
Small Rural	3	0	0	0	
**Years since completed training**					<0.001
<5	89	7	0	0	
5 to 9	459	38	1	0.3	
10 to 19	598	49	10	3	
≥20	73	6	389	97	
**Primary specialty, pediatric emergency medicine**	1,146	94	201	50	<0.001

© 2021 Bennett CL et al. *JAMA Network Open.*

Abbreviations: EM (emergency medicine), international medical graduate (IMG), interquartile range (IQR), metropolitan statistical area (MSA), and United States (US).

[a]Excludes pediatric emergency physicians with pediatric emergency medicine board certification by training with other training (internal medicine, n=6) or no training (n=14)

© 2021 Bennett CL et al. *JAMA Network Open.*
